# Influence of light intensity distribution characteristics of light source on measurement results of canopy reflectance spectrometers

**DOI:** 10.1186/s13007-021-00804-8

**Published:** 2021-10-16

**Authors:** Hongfeng Yu, Yongqian Ding, Huanliang Xu, Xueni Wu, Xianglin Dou

**Affiliations:** 1 College of Engineering, Nanjing Agricultural University, Nanjing, 210031 China; 2 College of Artificial Intelligence, Nanjing Agricultural University, Nanjing, 210031 China; 3 Collaborative Innovation Center for Modern Crop Production co-sponsored by Province and Ministry, Nanjing, 210095 China

**Keywords:** Canopy reflectance spectrometer, Irradiation characteristics, GreenSeeker, NDVI

## Abstract

**Background:**

The characteristics of light source have an important influence on the measurement performance of canopy reflectance spectrometer. The size of the effective irradiation area and the uniformity of the light intensity distribution in the irradiation area determine the ability of the spectrometer to express the group characteristics of the measured objects.

**Methods:**

In this paper, an evaluation method was proposed to theoretically analyze the influence of the light intensity distribution characteristics of the light source irradiation area on the measurement results. The light intensity distribution feature vector and the reflectance feature vector of the measured object were constructed to design reflectance difference coefficient, which could effectively evaluate the measurement performance of the canopy reflectance spectrometer. By using self-design light intensity distribution test system and GreenSeeker RT100, the evaluation method was applied to evaluate the measurement results.

**Results:**

The evaluation results showed that the vegetation indices based on the arithmetic average reflectance of the measured object could be obtained theoretically only when the light intensity distribution of the light source detected by the spectrometer was uniform, which could fully express the group characteristics of the object. When the light intensity distribution of the active light source was not uniform, the measure value was difficult to fully express the group characteristics of the object. And the measured object reflectance was merely the weighted average value based on the light intensity distribution characteristics.

**Conclusions:**

According to the research results of this paper, sunlight is the most ideal detection light source. If the passive light source spectrometer can improve the measurement method to adapt to the change of sunlight intensity, its measurement performance will be better than any active-light spectrometer.

## Background

Vegetation indices are the most commonly used data form to obtain crop growth parameters in agricultural remote sensing research [1]. Through regression analysis and other methods, these vegetation indices can establish the corresponding empirical inversion models of crop growth information (such as leaf area index [2, 3], biomass [4, 5], nitrogen [6, 7], chlorophyll [8, 9], plant diseases and pest recognition [10, 11], etc.). At present, the traditional detection methods of crop nutritional status (nitrogen content) mainly include: Kjeldahl method [12] and Dumas combustion method [13], which are destructive and practical indoor measurement methods. The obstacles of the traditional detection methods lie in long digestion time, many testing links, and the use of dangerous chemical reagents. For batch samples, the detection task is heavy, time-consuming, laborious, and easy to cause environmental pollution. Compared with traditional biochemical methods, the monitoring technology of crop canopy reflectance spectrometers has the advantages of fast, non-destructive, no space–time limited, and has become an important means to obtain crop growth information [14, 15]. Therefore, more and more scholars consider using crop canopy reflectance spectrometer with specific bands to obtain specific vegetation indices and corresponding crop growth parameters.

In this study, the crop canopy reflectance spectrometer for NDVI was used for research object. According to whether they have their own light sources, crop canopy reflectance spectrometers can be divided into active light source type and passive light source type. The early developed reflectance spectrometers mostly used passive light source, that was, the spectrometers used sunlight as the measure light source. The advantage of sunlight as the measure light source is that the light of the measured object is uniform, and the reflectance spectrometer can truly reflect the growth status of crop canopy. Typical products such as SRS (produced by Meter group, Inc. USA) can be used [16]. However, the biggest limitation of the passive light source reflectance spectrometers is that the measurement is affected by the environment and weather, and the low light irradiation conditions (night, rainy days, etc.) cannot be used. In addition, due to the changes of solar intensity and solar altitude angle, the measurement results of spectrometer will also be unstable [17]. Therefore, the following commercial crop reflectance spectrometers mainly use active light sources. The commercialized active light source crop reflectance spectrometers mainly include GreenSeeker handheld reflectance spectrometer [18] produced by N-tech Industries Inc., USA, and Cropcircle ACS-470 handheld reflectance spectrometer [19] produced by Holland scientific company. The structure and principle of Greenseeker and Cropcircle are similar. The light source adopts small emission angle (no more than 60 degrees), medium power narrow band LED (single LED luminous power is less than 1 W). The light source is shaped by a lens to form an illumination area of a specific shape (rectangular or linear). The reflected light intensity is detected by the photosensitive sensor. The effective measure height range is 60–160 cm, which is mainly used for handheld measure [20].

In fact, the VIs measurement method of passive light source spectrometers is based on the reflectance of the crop canopy, while the reflectance measurement needs white board correction when the light intensity changes. It results in poor adaptability to the light environment and inconvenience to use. However, it needn't to know the reflectance value of the specific detection wavebands for the active light source spectrometers, but only needs to obtain the relative intensity between them because the transmission power of each detection band is stable, this ensures that the measurement results of active light source spectrometers are not affected by the change of measurement height. That is why the active light sources spectrometers take more popularity in commerce, such as GreenSeeker, Crop Circle, LeafSpec and Cropspec TM etc. Sunlight is an ideal light source. The uniform intensity distribution and the unlimited covering size of detection area are the incomparable advantages of the sunlight to the active light source. The traditional method based on passive light source for measuring vegetation indices (VIs) cannot make full use of the advantages of the sunlight. At present, taking the property of the constant proportion of solar radiation energy in each component waveband, a new calculation method of VIs (in form of ratios) using sunlight as light source was put forward. This method can not only make full use of the advantages of the sunlight, such as uniform irradiation area and wide measurement range, but also obtain the characteristics similar as the active light source spectrometers that the measuring results had strong adaptability to light intensity and measurement heights and no whiteboard correction is needed in application. It may provide another solution for measuring VIs with passive light source and encourage people to develop new kind of passive light source spectrometers [21].

In order to reflect the canopy reflectance as truly as possible, the performance of the detection light source of crop canopy reflectance spectrometer is required, that is, the light intensity distribution of the detection light source is uniform. Hence, the design of optical system is the core content of the canopy reflectance spectrometer. It is pointed out that the uniformity of light intensity in the irradiation area after shaping is the key to the design of optical system. Due to the differences of canopy structure (such as leaf inclination, leaf density, canopy height) and nutritional status, the reflectance of actual canopy will be different. If the light intensity in the irradiation area of the light source is not uniform, the measure signal of the reflectance spectrometer will not be able to effectively obtain the average reflectance characteristics in the measure area, but only some weighted characteristics related to the light intensity distribution characteristics, which will lead to inaccurate vegetation indices and agronomic parameters. However, there is a lack of a unified evaluation method to evaluate the impact of light source on spectral data, which limits the improvement of crop canopy reflectance spectrometer or light path system.

In this paper, an evaluation method was proposed to theoretically analyze the influence of the light intensity distribution characteristics of the light source irradiation area on the measurement results. The light intensity distribution feature vector and the reflectance feature vector of the measured object were constructed to design reflectance difference coefficient, which could effectively evaluate the measurement performance of the canopy reflectance spectrometer. By using self-designed test system, the evaluation method was applied to evaluate the measurement results based on passive light source and GreenSeeker RT100. This method can be a new way for evaluating the performance of canopy reflectance spectrometers, and provide a possible way to improve the performance of active light source spectrometers.

## Materials and methods

In this section, the performance evaluation method based on reflectance analysis was proposed and the specific scheme of using performance evaluation method for crop canopy reflectance spectrometer was described.

### The principle of performance evaluation method

Reflectance is the basic parameter to calculate NDVI [22], RVI [23] and other ratio type vegetation indices [24]. The basic purpose of reflectance measure for crop canopy is to obtain the group reflectance characteristics in the measure area, and the average reflectance in the region is an ideal index to reflect the group characteristics. Therefore, the overall reflectance $R$ measured by the spectrometer can represent the average reflectance $\overline{R}$ of each block area in the measure area, that is, the following formula (1) is expected to hold:

1 $$R = \frac{{c_{1} + c_{2} + ... + c_{n} }}{{b_{1} + b_{2} + ... + b_{n} }} = \overline{R}$$

where $\overline{R} = \frac{1}{n}\sum\nolimits_{i = 1}^{n} {R_{i} } = \frac{1}{n}\sum\nolimits_{i = 1}^{n} {\frac{{c_{i} }}{{b_{i} }}}$, $b_{1} ,b_{2} ,.......,b_{n}$ were the standard whiteboard reflectance response value detected by the spectrometer in each block, and $c_{1} ,c_{2} ,.......,c_{n}$ were the canopy reflectance response value detected by the spectrometer in each block.

The reflectance ratio coefficient $\{ k_{i} \}$ was introduced to express the relationship between the average reflectance and the reflectance of each block of the canopy. If the reflectance of the spectrometer to the block area $i$ was expressed as $R_{i} { = }k_{i} \overline{R}$, then the canopy reflectance $R$ can be expressed as formula (2).

2 $$R = \overline{R} \frac{{k_{1} b_{1} + k_{2} b_{2} + ... + k_{n} b_{n} }}{{b_{1} + b_{2} + ... + b_{n} }}$$

In this study, $\vec{b}$ represents vector ${\{b_{1} ,b_{2} ,\ldots,b_{n}\} }$, $\vec{k}$ represents vector ${\{ k_{1} ,k_{2} ,\ldots,k_{n} \}}$, $\vec{I}$ represents vector $\{ 1,1,.......,1\}$, and $\theta_{k - b}$,$\theta_{I - b}$, and $\theta_{k - I}$ are used to represent the angles between $\vec{k}$ and $\vec{b}$, $\vec{I}$ and $\vec{b}$, $\vec{I}$ and $\vec{k}$, respectively. Since the components of each vector contain positive components, the range of included angle was $[0,90^{^\circ } ]$. Then formula (2) can be expressed as formula (3).

3 $$R = \overline{R} \frac{{\vec{b} \cdot \vec{k}}}{{\vec{b} \cdot \vec{I}}} = \overline{R} \frac{{\left\| {\vec{b}} \right\| \cdot \left\| {\vec{k}} \right\|\cos (\theta_{k - b} )}}{{\left\| {\vec{b}} \right\| \cdot \left\| {\vec{I}} \right\|\cos (\theta_{I - b} )}} = \overline{R} \frac{{\left\| {\vec{k}} \right\|}}{\sqrt n }\frac{{\cos (\theta_{k - b} )}}{{\cos (\theta_{I - b} )}}$$

Similarly, if formula (1) holds, then formula (4) below holds:

4 $$R = \frac{1}{n}\sum\limits_{i = 1}^{n} {R_{i} } { = }\frac{1}{n}\sum\limits_{i = 1}^{n} {\frac{{c_{i} }}{{b_{i} }}} { = }\frac{{\overline{R} }}{n}\sum\limits_{i = 1}^{n} {k_{i} } { = }\frac{{\overline{R} }}{n}\vec{k} \cdot \vec{I} = \frac{{\overline{R} }}{n}\left\| {\vec{k}} \right\| \cdot \left\| {\vec{I}} \right\|\cos (\theta_{k - I} ) = \overline{R} \frac{{\left\| {\vec{k}} \right\|}}{\sqrt n }\cos (\theta_{k - I} )$$

The problem of proving the validity of formula (1) can be transformed into that of formula (5).

5 $$\frac{{\cos (\theta_{k - b} )}}{{\cos (\theta_{I - b} )}} = \cos (\theta_{k - I} )$$

When the angle between vector $\vec{k}$ and vector $\vec{I}$ is 0° or vector $\overrightarrow {b}$ and vector $\vec{I}$ is 0°, formula (5) obviously holds, which is equivalent to that the reflectance is the same everywhere in the measure area, or the light intensity distribution is uniform in the measure range.

When the angle between vector $\vec{k}$ and vector $\vec{I}$ is not 0° or vector $\overrightarrow {b}$ and vector $\vec{I}$ is not 0°, that is to say, formula (5) may not be true, and the degree of difference is related to the characteristics of vector $\vec{k}$ and vector $\overrightarrow {b}$.

The reflectance difference coefficient $E$ was introduced as the performance evaluation method to characterize the difference between the average reflectance and the measured reflectance, which could be calculated according to formula (6).

$$E = |\frac{{p_{1} }}{{p_{2} }} - 1|{\text{*100\% }}$$

where,

$$p_{1} = \frac{{\cos (\theta_{k - b} )}}{{\cos (\theta_{I - b} )}},\;p_{2} = \cos (\theta_{k - I} )$$

In general, the reflectance of each block of the measured object is always different. In theory, the average reflectance of the measured object can be obtained only if the light intensity distribution of the measure light source is uniform, and the group characteristics of the measured object can be fully expressed. The measure light source of active light source spectrometer is difficult to achieve uniform light intensity distribution, in fact, it is difficult to fully express the group characteristics of the object, while the passive light source spectrometer, whose measure light source is sunlight, has a very superior uniform light intensity distribution characteristic. In theory, its measured value can fully express the group characteristics of the object under test. However, due to the defects of current measure methods, there are many factors affecting the measure results of passive light source spectrometers, and it is not as convenient as the active light source spectrometer.

### The scheme of performance evaluation for canopy reflectance spectrometers

In order to verify the performance evaluation method, the following four methods were proposed for NDVI acquisition, which involved two different light intensity distribution characteristic vectors $\vec{b}$. When the reflectance ratio coefficient vector $\overrightarrow {k}$ and light intensity distribution characteristic vector $\vec{b}$ are known, the above evaluation method can be used to analyze the differences among them qualitatively and quantitatively.

#### Definition of four NDVI acquisition methods

The following four methods were proposed for NDVI acquisition, and the corresponding NDVI results of each method were represented by different symbols as follows:

$N_{r}$: The NDVI value was calculated according to the average reflectance of the visible and near-infrared bands of the test object.

$N_{m}$: NDVI measure value of test object was measured by the reflectance spectrometer.

$N_{t}$: The NDVI value was calculated based on the weighted reflectance of the test object obtained from the light intensity distribution characteristics of the active light source of the spectrometer.

$N_{s}$: Under the condition of sunlight irradiation, the NDVI value was calculated by obtaining the actual reflectance of the test bands of the measured object.

According to the above definition, the four NDVI acquisition methods involve two different light intensity distribution characteristic vectors $\vec{b}$, which are the light intensity distribution vector of the active light source and the light intensity distribution vector of the solar irradiation area. When the reflectance ratio coefficient vector $\overrightarrow {k}$ and light intensity distribution characteristic vector $\vec{b}$ are known, the above method can be used to analyze the differences among $N_{r}$, $N_{m}$, $N_{t}$ and $N_{s}$ qualitatively and quantitatively.

#### The description of the portable test system for measuring the light intensity distribution

**Fig. 1:**
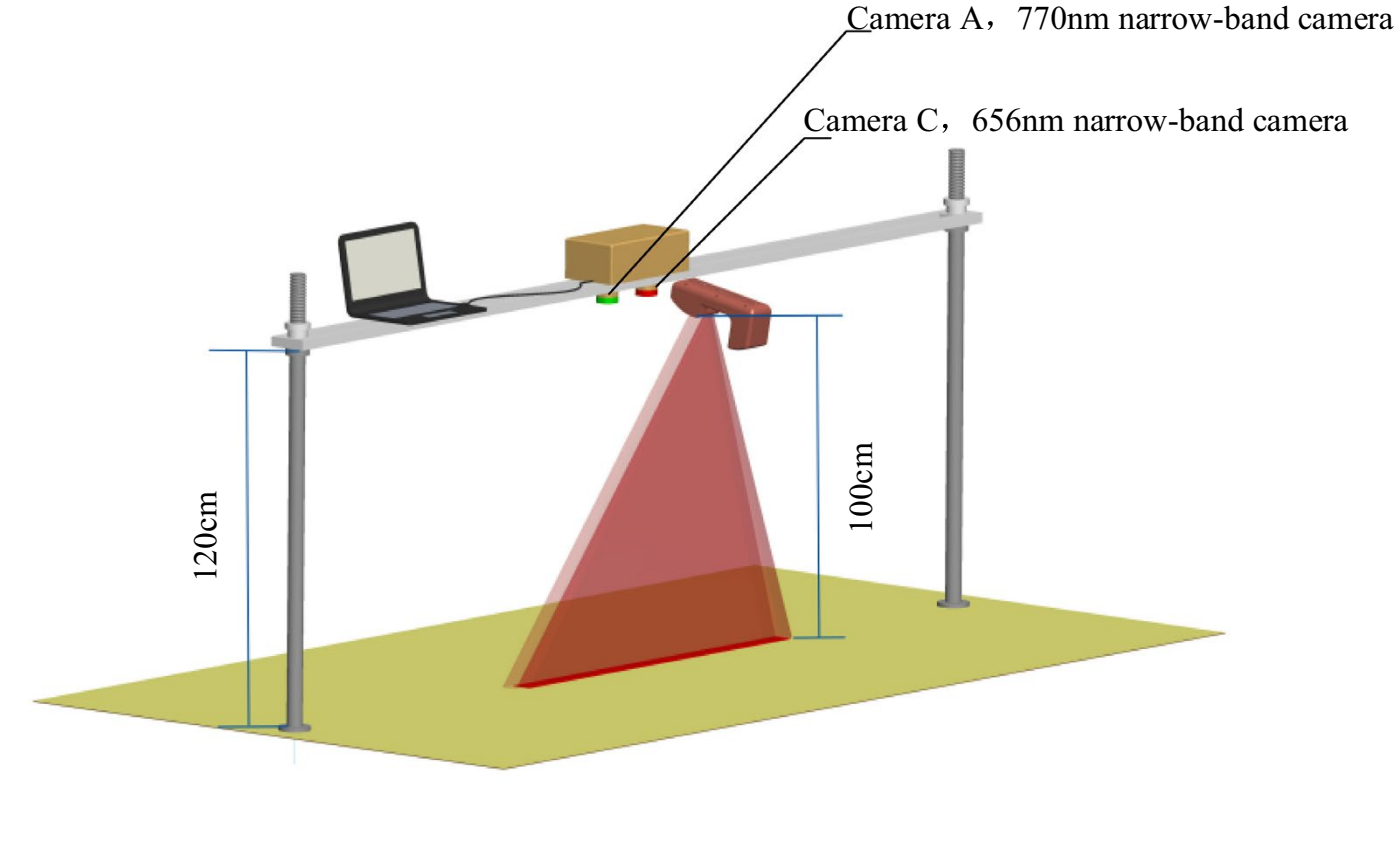
Schematic diagram of light intensity distribution test system

In order to measure the light intensity distribution characteristics of the canopy reflectance spectrometer, two industrial cameras with narrow-band filter lens [25, 26] were used to construct a test system as shown in Fig. 1. The specific parameters of the test system were shown in Table 1.

**Table 1:** Specific parameters of light intensity distribution test system for light strip

Device names	Manufacturer and model	Performance parameters
Industrial camera	The model WP-UC600 made by the company named by Huangudongli in China	Resolution: 3072*2048; Frame rate: 30 FPS; Sensor: 2 / 3 inch color CMOS; Spectral response range:380–1100 nm
Camera lens	The model WP-5M0825-C made by the company named by Huangudongli in China	Focal distance: 8 mm; FOV(Field of view angle): 57.6*49.3*71.2 degree
Optical filter	Absorption type narrow band filter made by the company named by Gengxu Optoelectronics in China	Central wavelength:656 nm and 770 nm; Bandwidth:: 20 nm
Canopy reflectance spectrometer	GreenSeeker RT100	Emission wavelength: Near infrared band: 770 nm; red band: 656 nm Range of NDVI measure: 0.00–0.99; Range of voltage output: 0.00–0.99 V Measuring area size: 24 inches ± 4 inches;

#### Test design for light intensity distribution characteristics measure

The test for measuring the intensity distribution characteristics included two steps. The first step was to determine the effective measure area of GreenSeeker RT100, and the second step was to calculate the intensity distribution characteristics in effective measure area.

##### Determination of effective measure area of GreenSeeker RT100

In theory, if the light intensity distribution feature vector and the reflectance feature vector can be calculated, the evaluation for canopy reflectance spectrometers can be applied in the actual measure conditions. However, because the reflectance of canopy in the wild is very difficult to measure, the reflectance feature vector needs to be measured by specialized instrument, such as hyperspectral spectrometers, in special artificial light environment. As we know, the canopy reflectance measurement with hyperspectral spectrometers in a large-scale area and wild environment is something impractical.

**Fig. 2:**
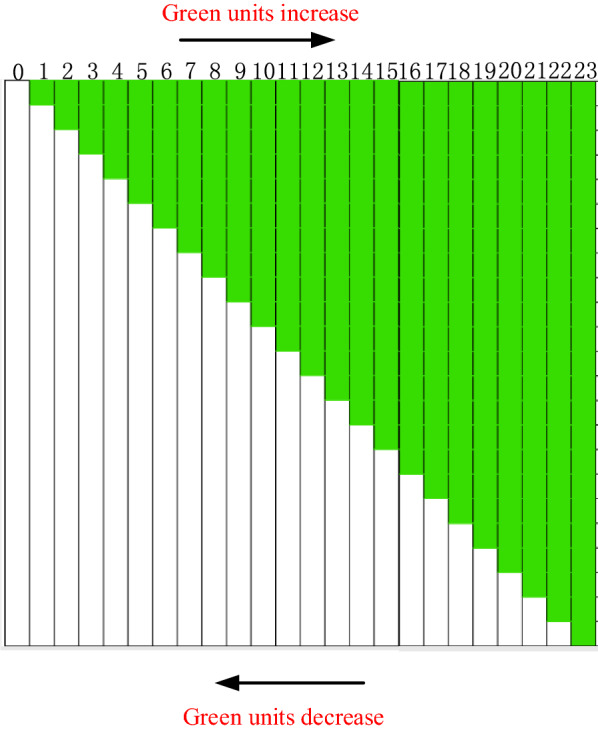
Schematic diagram of test strips

In order to determine the effective measure area of GreenSeeker RT100, we designed 23 test strips with fixed reflectance characteristics, as shown in Fig. 2. The size of each test strip was 115 cm × 5 cm, which could be divided into 23 units. Each unit was set as a white or green color block with fixed reflectance. After the high-resolution spectrometer (model Imspector V-10E, made by Specim company in America) measure, the reflectance of the white block at 656 and 770 nm were 0.7641 and 0.7743, and that of the green block were 0.1412 and 0.6645, respectively.

During the test, each test strip was placed under the active light source of GreenSeeker RT100, and it was symmetrical about the vertical line of the light source center. The measure height of GreenSeeker RT100 was 100 cm. The test time was 8:00–10:00 a.m., in Nanjing, Jiangsu province, China, October 9, 2020. The weather was sunny, and the variation range of external light intensity was about 80,000 lx–140000 lx. After each test, the NDVI measure value was recorded manually.

In the test process, the test strips were tested one by one according to the sequence of increasing green units shown in Fig. 2, and then the test strips were tested one by one according to the decreasing order of green units.

##### Light intensity distribution characteristics of effective measure area

According to the above test results of the effective measure area, the effective measure area with white matte paper (the reflectance of 656 and 770 nm was 0.7641 and 0.7743 respectively) was placed directly below the industrial cameras of the test system. It was used to collect the strip images of irradiation area formed by GreenSeeker RT100 at 100 cm measure height and the light strip images of sunlight in the same irradiation area. The light strip of GreenSeeker RT100 irradiation area was collected in dark room, while the solar radiation area light strip was collected in the outside sunlight condition. The test time of the solar light strips was 10:00–12:00 a.m., in Nanjing, Jiangsu province, China, October 10, 2020. The weather was sunny, and the variation range of external light intensity was about 90,000 lx–140000 lx.

#### Test design for four NDVI acquisition methods

##### The Acquisition of reflectance ratio coefficient vectors

**Fig. 3:**
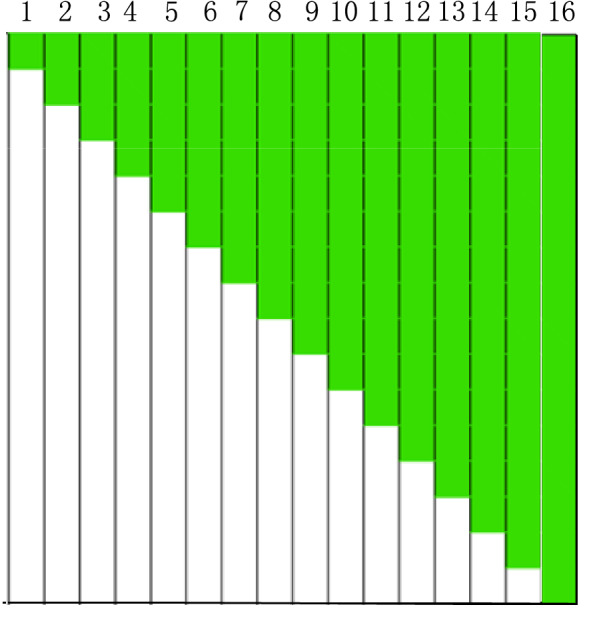
The test strips for NDVI measurement

In order to study the influence of different light intensity distribution characteristics on NDVI measurement, the test strips with fixed reflectance distribution characteristics similar to Fig. 2 were designed. According to the measure results of GreenSeeker RT100 effective measurement area, there were 16 test strips in total, each of which was 80 cm × 5 cm in size, and was divided into 16 5 cm × 5 cm units, as shown in Fig. 3. Because the reflectance of each unit of the test strip represents each component of the reflectance ratio coefficient vector, the reflectance ratio coefficient vectors $\vec{k}_{{6{56}}}$ and $\vec{k}_{{{77}0}}$ of each test strip can be obtained.

##### The Acquisition of $N_{m}$ and $N_{s}$

During the test, each test strip was placed in the irradiation area of GreenSeeker RT100, and the center of the strip coincided with the center of the irradiation area. The measurement height of GreenSeeker RT100 was 100 cm. The GreenSeeker RT100 NDVI measurement values of 16 test strips in Fig. 3 were obtained in turn, which represented $N_{m}$.

$N_{s}$ was obtained under the condition of sunlight by using the device in Fig. 1. During the test, the center position of the test strip in Fig. 3 coincided with the center of the two narrow-band cameras in Fig. 1, and then the narrow-band images of all the test strips in 656 and 770 nm bands in Fig. 3 were synchronously obtained by using the device in Fig. 1.

At the same time, in order to obtain the reference value of the standard whiteboard, a white reference test strip similar to the test strip No. 0 in Fig. 2 was placed next to each test strip, and the average gray value of the reference test strip was divided by its actual reflectance as the reflectance reference value of the standard whiteboard. The calculation of $N_{s}$ was shown in formula (7).

7 $$N_{s} { = }\frac{{\frac{{P_{770} }}{{P_{770\_w} }} - \frac{{P_{{6{56}}} }}{{P_{{6{56}\_w}} }}}}{{\frac{{P_{770} }}{{P_{770\_w} }} + \frac{{P_{{6{56}}} }}{{P_{{6{56}\_w}} }}}}$$

where,

$P_{770}$_and_ $P_{{{656}}}$ represented the average gray value of the test strip image in 770 and 656 nm band, respectively.

$P_{770\_w}$ and $P_{{{656}\_w}}$ represented reflectance reference value of standard whiteboard in 770 and 656 nm band, respectively.

The test was conducted from 10:00 am to 11:00 am on October 16, 2020, in Nanjing, Jiangsu Province, China. The weather was fine and solar irradiation intensity varied approximately from 80000 to 160000 lx.

##### Calculation method of $N_{t}$ and $N_{r}$.

According to the definition in Sect. 2.2.1, the calculation method of $N_{t}$ and $N_{r}$ in this test scheme can be expressed in the form of formulas (8) and (9).

$$N_{r} = \frac{{M*(R_{770\_g} - R_{{6{56}\_g}} ) + (N - M)*(R_{770\_w} - R_{{6{56}\_w}} )}}{{M*(R_{770\_g} + R_{{6{56}\_g}} ) + (N - M)*(R_{770\_w} + R_{{6{56}\_w}} )}}$$

$$N_{t} = \frac{{\frac{{R_{770\_w} }}{{|\overrightarrow {{b_{770} }} |}}*\sum\limits_{i = 1}^{M} {b_{i\_770} } + \frac{{R_{770\_g} }}{{|\overrightarrow {{b_{770} }} |}}*\sum\limits_{i = M}^{N} {b_{i\_770} } - \frac{{R_{{6{56}\_w}} }}{{|\overrightarrow {{b_{{6{56}}} }} |}}*\sum\limits_{i = 1}^{M} {b_{{i\_6{56}}} } - \frac{{R_{{6{56}\_g}} }}{{|\overrightarrow {{b_{{6{56}}} }} |}}*\sum\limits_{i = M}^{N} {b_{{i\_6{56}}} } }}{{\frac{{R_{770\_w} }}{{|\overrightarrow {{b_{770} }} |}}*\sum\limits_{i = 1}^{M} {b_{i\_770} } + \frac{{R_{770\_g} }}{{|\overrightarrow {{b_{770} }} |}}*\sum\limits_{i = M}^{N} {b_{i\_770} } + \frac{{R_{{6{56}\_w}} }}{{|\overrightarrow {{b_{{6{56}}} }} |}}*\sum\limits_{i = 1}^{M} {b_{{i\_6{56}}} } + \frac{{R_{{6{56}\_g}} }}{{|\overrightarrow {{b_{{6{56}}} }} |}}*\sum\limits_{i = M}^{N} {b_{{i\_6{56}}} } }}$$

where $M$ represented the number of green units in test strip; $N$ represented the number of total units in test strip; $R_{770\_w}$ and $R_{{6{56}\_w}}$ represented the reflectance value of the white color block in 770 nm and 656 nm band, respectively.

$R_{770\_g}$ and $R_{{6{56}\_g}}$ represented the reflectance value of the green color block in 770 nm and 656 nm band, respectively.

$\overrightarrow {{b_{770} }}$ and $\overrightarrow {{b_{{6{56}}} }}$ represented GreenSeeker RT100 light intensity distribution characteristic vectors of 770 nm and 656 nm band, respectively.

## Results and analysis

In this part, the GreenSeeker RT100 reflectance spectrometer was taken as the test object, and the performance evaluation method proposed in this paper was applied to analyze the influence of the light intensity distribution characteristics of the measured light strip and the reflectance distribution characteristics of the measured object on the NDVI measure results. The remainder of the section is organized as follows: “Measure results of light intensity distribution characteristics” section shows the results of the active and solar light intensity distribution characteristics, and “The NDVI results of the four acquisition methods” section shows the results of the four NDVI acquisition method. “The performance evaluation method for analyzing the four NDVI acquisition methods” section presents the performance evaluation method for analyzing the four NDVI acquisition methods.

### Measure results of light intensity distribution characteristics

#### The effective irradiation area of GreenSeeker RT100

**Fig. 4:**
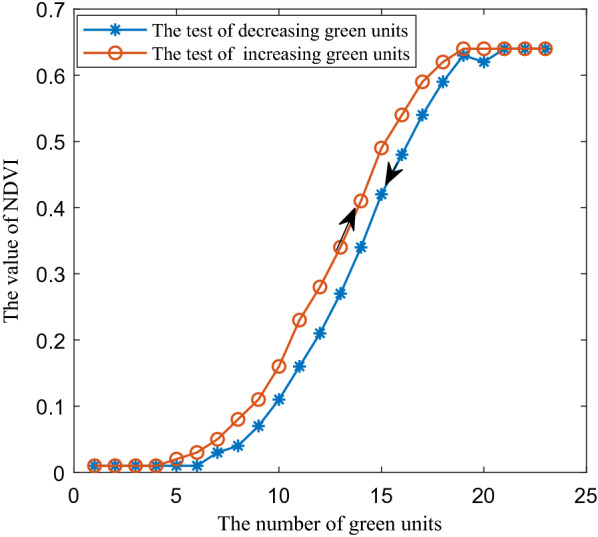
NDVI results of test strips measured by GreenSeeker RT100

Figure 4 showed the NDVI test results of test strips with different serial numbers. When the green units of the test strips were less than 5, the NDVI value was basically constant at 0.01, while the green units were greater than 20, the NDVI value was basically constant at 0.64. When the number of green units of the test strip was 5 ~ 20, the NDVI value had obvious change. Therefore, the effective measure area length of GreenSeeker RT100 at 100 cm measure height was about 80 cm which was covered by 16 color units in the middle of each test strip.

**Fig. 5:**
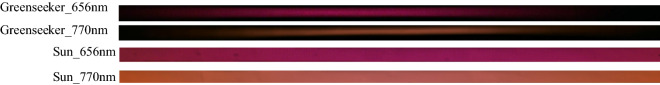
light strip image of effective measure area

#### The test results of light intensity distribution characteristics

According to the above test results, the whiteboard area of 5 cm * 80 cm directly below the GreenSeeker RT100 in the Fig. 1 was used as the effective measure area. The effective measure area light strip images of sunlight and GreenSeeker RT100 active light source collected by narrow-band cameras are shown in Fig. 5.

**Fig. 6:**
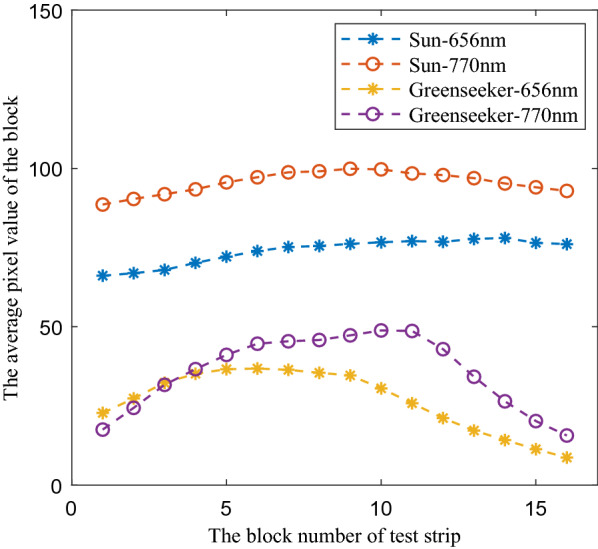
The light intensity distribution of sunlight and GreenSeeker RT100in effective measure area

According to the characteristic that the gray value of the camera was proportional to the incident light intensity, firstly, the RGB images captured by the industrial cameras were converted into gray images, and the gray values were taken as the characterization of the light intensity distribution. Then, the light strip gray images were divided into 16 5 cm × 5 cm regions according to the length direction, and the pixel gray average value of each region was calculated. The pixel average value vector of the 16 regions was the light intensity distribution vector $\overrightarrow {b}$. The light intensity distribution of sunlight and GreenSeeker RT100 light strips is shown in Fig. 6.

The uniformity of light intensity distribution can be evaluated by the variation coefficient of the light intensity in each block area of light strip. The specific results were as follows: under the sunlight condition, the light intensity variation coefficients of 656 and 770 nm were 5.42 and 3.63%, respectively. Under the condition of GreenSeeker RT100 active light source, the light intensity variation coefficients of 656 and 770 nm were 36.72 and 36.79%, respectively.

Because the area closer to the center axis of the camera lens has a larger reflection angle, which can make the camera produce a relatively strong response value, the measured light intensity distribution itself will show the phenomenon of large response value in the middle and small response value in the two ends. This is the inevitable measure error caused by the response characteristics of the camera in the measure system, but the overall results can still reflect the difference of the light intensity distribution of the measure light strip itself. According to the coefficient of variation, the uniformity of light intensity in the solar irradiation area was obviously better than that in the GreenSeek RT100 irradiation condition.

### The NDVI results of the four acquisition methods

**Fig. 7:**
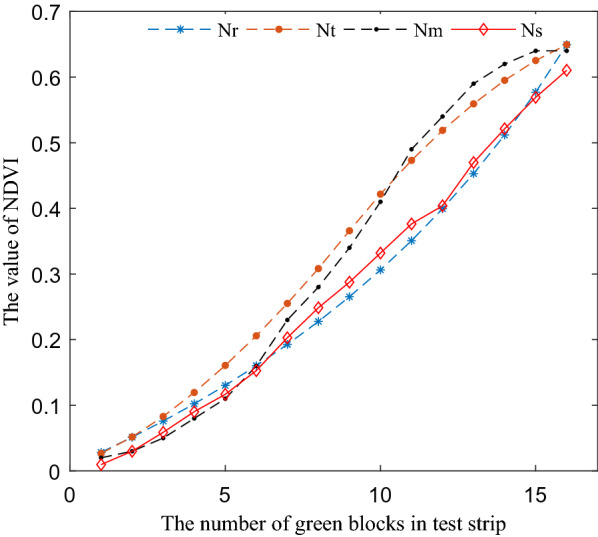
NDVI results of the test strips corresponding to different acquisition methods

Figure 7 showed the test results of four NDVI acquisition methods. In Fig. 7, the test results can be obviously divided into two groups according to the degree of data proximity.$N_{s}$ and $N_{r}$ can be divided into one group, the maximum absolute deviation value was 0.0391, the average value was 0.0171, and the standard deviation value was 0.0088. The test results of $N_{m}$ and $N_{t}$ can be divided into another group, the maximum absolute deviation value was 0.0507, the average value was 0.0254, and the standard deviation value was 0.0125.

**Fig. 8:**
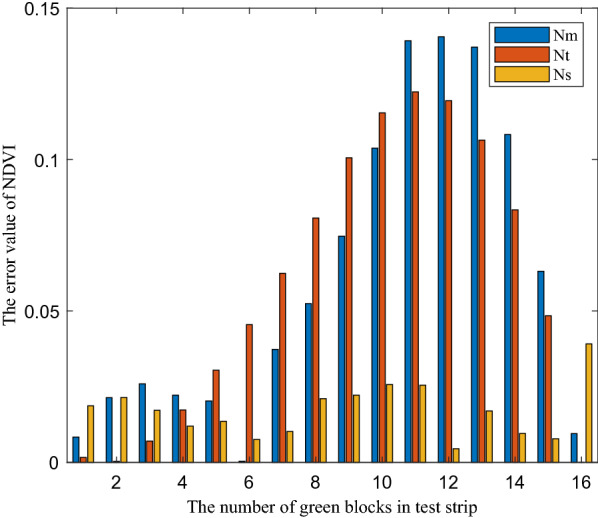
The absolute error value of $N_{t}$ and $N_{m}$ and $N_{s}$, respectively

However, there were obvious differences between the two groups of the test data. Taking $N_{r}$ as reference, the absolute error values of $N_{t}$,$N_{m}$ and $N_{s}$ were shown in Fig. 8. The maximum absolute error value of $N_{t}$ and $N_{r}$ was 0.1223, the average value was 0.0588, and the standard deviation value was 0.0459. The maximum absolute error value of $N_{m}$ and $N_{r}$ was 0.1405, the average absolute error value was 0.0603, and the standard deviation value of absolute error was 0.0505.

### The performance evaluation method for analyzing the four NDVI acquisition methods

**Fig. 9:**
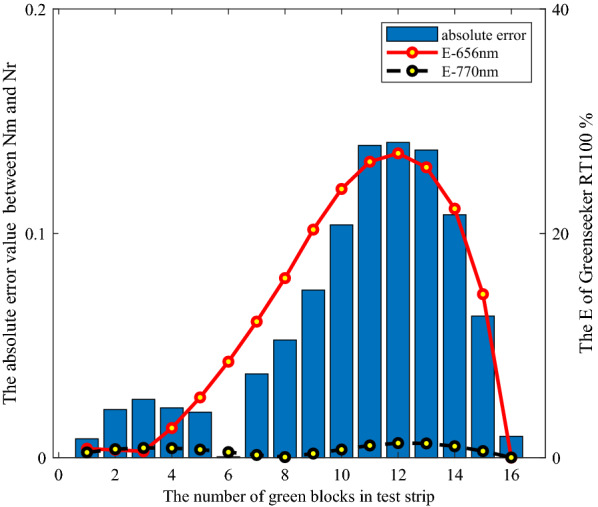
Relationship between absolute value of NDVI error and two band reflectance difference coefficients under irradiation condition of GreenSeeker RT100

**Fig. 10:**
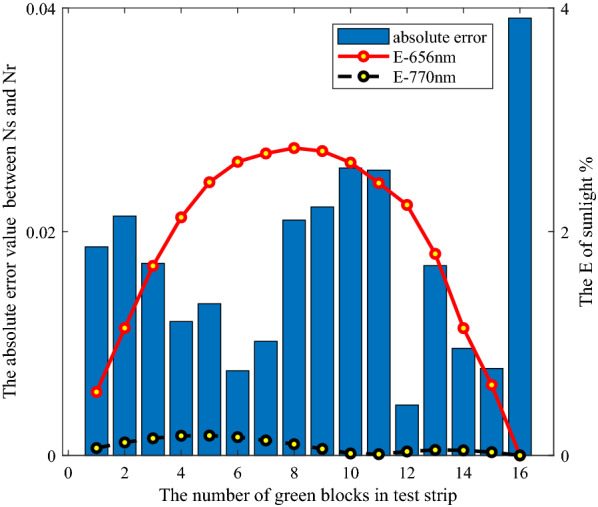
Relationship between absolute value of NDVI error and two band reflectance difference coefficients under solar irradiation conditions

The performance evaluation was used to analyze and discuss the results of the four NDVI acquisition methods. Figures 9 and 10 showed the relationship between the absolute error value of NDVI and the two bands reflectance difference coefficients $E$ under the irradiation conditions of GreenSeeker RT100 and sunlight, respectively.

(1) When the light intensity distribution was not uniform or the reflectance of the measured area was different, the deviation between the weighted reflectance and the average reflectance of the measured area was determined by the light intensity distribution characteristics and the reflectance difference characteristics. The next two cases were discussed separately.

*In the case of reflectance difference* When the green color units gradually increased in the middle stage in Fig. 3, the NDVI values obtained by the four methods in Fig. 8 increased significantly. In this case, the relationship between the absolute error of $N_{m}$ and $N_{r}$ and the two band reflectance difference coefficients $E$ was illustrated as an example, as shown in Fig. 9. The results showed that with the increase of the green units of the test strips, the two band reflectance ratio coefficients $\vec{k}_{{}}$ changed significantly, and the corresponding two band reflectance difference coefficients $E$ also changed significantly.

*In the case of uneven light intensity distribution* In Fig. 9, the average reflectance difference coefficients $E$ of GreenSeeker RT100 at 656 and 770 nm were 12.95 and 0.6639% respectively. In Fig. 10, the average reflectance difference coefficients $E$ of 656 and 770 nm were 1.8515 and 0.0832%, respectively. The reflectance difference coefficient $E$ of sunlight was far less than that of GreenSeeker RT100 active light source, so the average absolute deviation of $N_{s}$ was also less than that of $N_{m}$.

(2) When the light intensity distribution was uniform or the reflectance difference was small, the deviation between the weighted reflectance and the average reflectance in the measurement area decreased.

*In the case of uniform light intensity distribution* Compared with the absolute error results of GreenSeeker RT100, although the green color units of the test strips increased gradually under the sunlight condition, and the reflectance also suffered from a significant change process, the absolute error values of $N_{s}$ were much smaller because the sunlight intensity was relatively uniform, which indicated that the reflectance difference of the tested object did not affect the measurement results when the illumination intensity of the test light source was uniform.

*In the case of small reflectance difference* Reflectance ratio coefficients of two bands were small in the beginning and end stages of green color block increase of test strips, and NDVI values obtained by the four methods were close. Especially at the end of the test, all blocks of strips were green, and $N_{t}$ and $N_{m}$ and $N_{r}$ were basically coincident, which showed that when the reflectance of the measured object was uniform, the uneven light intensity did not affect the measurement results.

In general, the results of the absolute value of NDVI error and two band reflectance difference coefficients $E$ in Figs. 9 and 10 showed that under the same test strip, the two band reflectance difference coefficients $E$ under the active light source condition were significantly greater than that under the sunlight condition. The reason why the data values of $N_{s}$ and $N_{r}$ were close was that the intensity distribution vector $\overrightarrow {{b_{{}} }}$ tended to the ideal state of uniform intensity distribution under the condition of sunlight, and $N_{s}$ measured by sunlight was consistent with the theoretical calculation results $N_{r}$. The difference between $N_{m}$ and $N_{r}$ values indicated that the uneven intensity distribution of the active light source had an impact on the measurement results, and the consistency of $N_{m}$ and $N_{t}$ data further indicated that the measurement results of GreenSeeker RT100 tended to be based on the weighted average reflectance affected by the intensity distribution of the measured area.

## Discussion

### The effect of performance evaluation method

The difference of the four NDVI acquisition methods indicated that the NDVI measurement results of active light source reflectance spectrometer was not calculated based on the arithmetic average reflectance in the measurement area, but based on the weighted average reflectance affected by the intensity distribution of the measurement area. Moreover, the more uneven the light intensity in the irradiation area was, the greater the difference between the weighted reflectance and the arithmetic average reflectance was. The average absolute error analysis in “The NDVI results of the four acquisition methods” section also verified the test results.

Meanwhile, the absolute error values of NDVI of the active light source were also significantly greater than that of the sunlight, which indicated that the evaluation method proposed in this paper can scientifically evaluate the irradiation characteristic error of the light source. However, because the evaluation method was based on reflectance, the absolute deviation of NDVI in Figs. 9 and 10 cannot be one-to-one corresponding to the reflectance difference coefficient of a single band, but was determined by the reflectance difference coefficient of two bands. In addition, due to the measurement characteristics of the measurement system in Fig. 2, the measurement led to a measurement system error. Especially in the beginning and end values of the green color block in Fig. 10, there was a large deviation, but this did not affect the scientificity of the evaluation method.

### Comparing the evaluation method with other methods

At present, the performance evaluation of spectrometer has become a research hotspot. The evaluation of the effects of light source [27], optical path [28], canopy structure [29] and other factors on the performance of crop canopy reflectance spectrometer has been carried out. From the current literature, the mainstream research focuses on the influence degree of factors on performance using the orthogonal test analysis method. The corresponding results are obtained by changing the factor levels and compared with the standard measuring instruments to determine whether the influencing factors are significant, which is different from the theoretical evaluation method established in this manuscript. The research emphasis of this manuscript is to explain why the factors, such as the light intensity distribution of light source, will influence the measurement performance. We hope provide a new way or technical program to improve the performance of the spectrometers.

## Conclusion and perspectives

In this paper, a method for analyzing the measurement performance of crop canopy reflectance spectrometer based on the irradiation characteristics of the light source was proposed. By constructing the light intensity distribution vector and canopy reflectance ratio coefficient vector, the measurement performance of canopy reflectance spectrometer can be effectively evaluated. The evaluation results showed that only when the light intensity distribution of the light source detected by the spectrometer was uniform, the vegetation indices based on the average reflectance of the measured object could be obtained in theory to fully express the group characteristics of the measured object. When the light intensity distribution of the active light source was not uniform, the measured value was difficult to fully express the group characteristics of the object. The deviation between the measure value and the vegetation indices calculated based on the average reflectance was determined by the light intensity distribution characteristics and the reflectance difference characteristics of the measured object.

The performance analysis of the canopy reflectance spectrometer also pointed out further application for improvement. On one hand, optimizing light path structure of active light source spectrometers to produce more uniform irradiation area will be efficient way to improve the measurement performance of the spectrometers. On the other hand, The uniform intensity distribution and the unlimited covering size of detection area are the incomparable advantages of the sunlight to the active light source. Researching new measurement method or principle, which can make full use of the advantageous properties of sunlight, may be a meaningful way to improve measurement performance of traditional passive light source spectrometers.

## Data Availability

The data sets used and/or analyzed during the current study available from the corresponding author on reasonable request.
